# Enhancement of Mechanical Properties and Porosity of Concrete Using Steel Slag Coarse Aggregate

**DOI:** 10.3390/ma13122865

**Published:** 2020-06-26

**Authors:** Md Jihad Miah, Md. Munir Hossain Patoary, Suvash Chandra Paul, Adewumi John Babafemi, Biranchi Panda

**Affiliations:** 1 Department of Civil Engineering, University of Asia Pacific, Dhaka 1205, Bangladesh; jihad.miah@uap-bd.edu (M.J.M.); 3dcometdesign@gmail.com (M.M.H.P.); 2 Department of Civil Engineering, International University of Business Agriculture and Technology, Dhaka 1230, Bangladesh; suvashpl@iubat.edu; 3 Department of Civil Engineering, Stellenbosch University, Private Bag X1, Matieland 7602, Stellenbosch, South Africa; ajbabafemi@sun.ac.za; 4 Department of Mechanical Engineering, Indian Institute of Technology Guwahati, Assam 781039, India

**Keywords:** steel slag aggregate, concrete, compressive and tensile strength, length change, porosity

## Abstract

This paper investigates the possibility of utilizing steel slags produced in the steelmaking industry as an alternative to burnt clay brick aggregate (BA) in concrete. Within this context, physical, mechanical (i.e., compressive and splitting tensile strength), length change, and durability (porosity) tests were conducted on concrete made with nine different percentage replacements (0%, 10%, 20%, 30%, 40%, 50%, 60%, 80%, and 100% by volume of BA) of BA by induction of furnace steel slag aggregate (SSA). In addition, the chemical composition of aggregate through X-ray fluorescence (XRF) analysis and microstructural analysis through scanning electron microscopy (SEM) of aggregates and concrete were performed. The experimental results show that the physical and mechanical properties of concrete made with SSA were significantly higher than that of concrete made with BA. The compressive and tensile strength increased by 73% when SSA fully replaced BA. The expansion of concrete made with SSA was a bit higher than the concrete made with BA. Furthermore, a significant lower porosity was observed for concrete made with SSA than BA, which decreased by 40% for 100% SSA concrete than 100% BA concrete. The relation between compressive and tensile strength with the porosity of concrete mixes are in agreement with the relationships presented in the literature. This study demonstrates that SSA can be used as a full replacement of BA, which is economical, conserves the natural aggregate, and is sustainable building material since burning brick produces a lot of CO_2_.

## 1. Introduction

In the utilization of industrial residues as construction raw materials, the concrete industry can play an important role in sustainable development, leading to considerable environmental benefits. Generally, aggregates (coarse and fine) occupy about 60–85% of the total volume of hardened concrete [1]. Aggregates are important constituents in the concrete composite that help to improve the various properties of concrete, including reducing the shrinkage and providing workability, volume stability, strength, and durability to the concrete [2]. In the numerous countries of South Asia, the concrete industry mostly depends on burnt clay brick aggregate (BA) due to the shortage of natural stones [3]. The production process of BA increases CO_2_ in the air, resulting in a large negative environmental impact and risk to human life. Though crushed stone aggregates are used in the concrete industry due to rapid urbanization, they are mostly imported from abroad, thereby, increasing the cost of concrete production. Due to the aforementioned issues, indeed, it is necessary to find possible alternative construction raw materials that can be used as coarse and fine aggregates in concrete construction works [4,5,6]. 

Steel slag is a byproduct of the steelmaking process which is produced during the separation of the molten steel from impurities in the steelmaking furnace, and it is used as coarse aggregate in concrete. It was found that steel slag aggregate (SSA) has superior physical and mechanical properties as well as lower carbon footprint and reduced negative environmental effects [7,8]. The compressive strength of concrete containing steel slag at 28 days was about 35% higher than the reference concrete [9]. The incorporation of different replacement percentages (15%, 25%, 50%, 75%, and 100%) of natural stone aggregate by SSA increased compressive and flexural strength, while it reduced the chloride ion permeability from 40% to 70% compared to concrete made with natural stone aggregate [10]. Similarly, research demonstrated that the physical and mechanical properties of concrete made of slag aggregate were higher as compared to natural aggregate [11]. Conversely, the reduction in workability was observed for concrete containing SSA compared to natural aggregate.

Netinger et al. [12] investigated the mechanical properties and corrosion resistance of concrete made with SSA. It was found that SSA can be used in concrete since it provides acceptable mechanical properties and no risk of corrosion of reinforcement. It was found that the carbonation treatments can significantly improve the strength and volume stability of concrete made with carbonated granulated steel slag aggregate [13]. Abu-Eishah et al. [14] observed that the concrete made with steel SSA provides high-strength compared to similar conventional concrete mixtures, which could be due to the strong bond between the cement/mortar matrix and SSA. It has been observed that concrete made with SSA decreased the workability of fresh concrete; it was claimed that SSA particles were angularly resulting in a reduction of the flowability of concrete [13]. Similarly, Qasrawi [15,16] observed that the addition of SSA decreased the workability of concrete and produced an unacceptable flow when more than 50% SSA were used. The reduction in a slump of concrete containing SSA was also observed by Sheen et al. [17]. 

Several studies have been carried out to investigate the mechanical performance of concrete made with SSA, but few focused on durability (e.g., porosity). To the authors’ knowledge, there seems to be no published work on concrete made with induction furnace SSA as a replacement of BA. This induction furnace slag is used mainly for landfills and sometimes as an alternative aggregate in road construction, which is not very common due to lack of research data. The lack of research data, limited information, and knowledge on the mechanical and durability performances of concrete made with SSA as a replacement for BA motivate this research work. Within this context, comprehensive experimental studies were conducted on the possibility of using induction furnace steel slag as coarse aggregate replacement for BA. This is with the goal to reduce the consumption of industrial-made brick (reduce CO_2_) and natural aggregate which is mainly imported from abroad (reduce the cost of concrete and obtain a sustainable construction material). The aim of this research work is to investigate the physical, mechanical (i.e., compressive and splitting tensile strength), length change, and durability (porosity) performances of concrete made with nine different replacement percentages (0%, 10%, 20%, 30%, 40%, 50%, 60%, 80%, and 100% by volume of BA) of BA by induction furnace SSA. To gain a deeper understanding of the role of SSA on the performance of concrete, the relation between strength (compressive and tensile) and porosity of concrete mixes was discussed. Additionally, the chemical composition of aggregate through X-ray fluorescence (XRF) analysis was determined and microstructural analysis using scanning electron microscopy (SEM) of aggregates and concrete was performed.

## 2. Experimental Methodology

### 2.1. Material Characterization

#### 2.1.1. Aggregates

**Figure 1:**
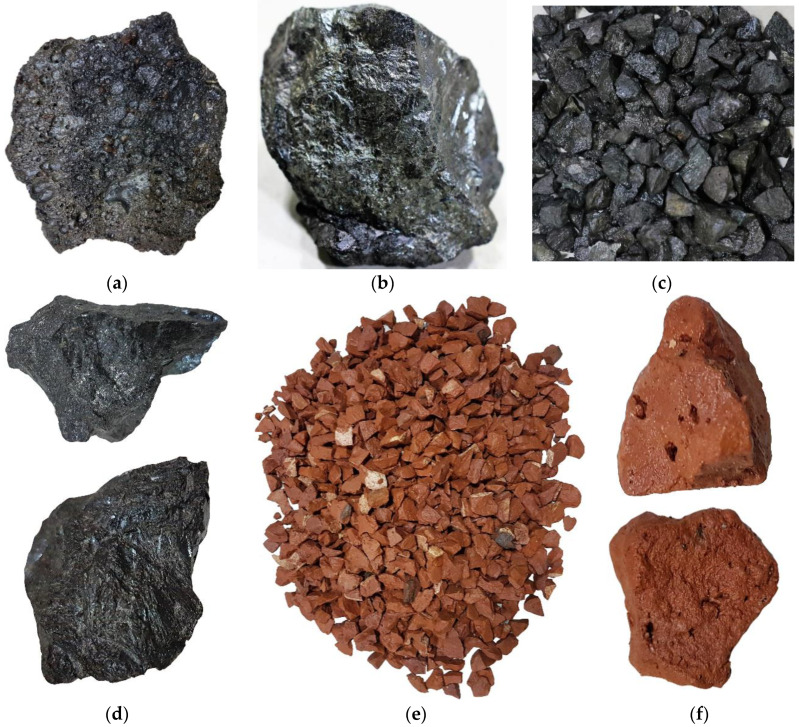
Induction furnace steel slag boulders: (**a**) steel slag with more void, (**b**) steel slag with less void; steel slag aggregate (SSA): (**c**) steel slag coarse aggregate, (**d**) close view of SSA; brick aggregate (BA): (**e**) brick coarse aggregate, (**f**) close view of BA.

Two types of coarse aggregates (first-class burnt clay brick and induction furnace SSA) were used in this study. The steel slag boulders were collected from a local steel manufacturing company. Two different types of steel slag boulders were found: some were light in weight with more voids (more porous, see Figure 1a), those were used in wastewater treatment [18], and others were denser with less voids (see Figure 1b). The SSAs with the denser structure were used in this study.

**Figure 2:**
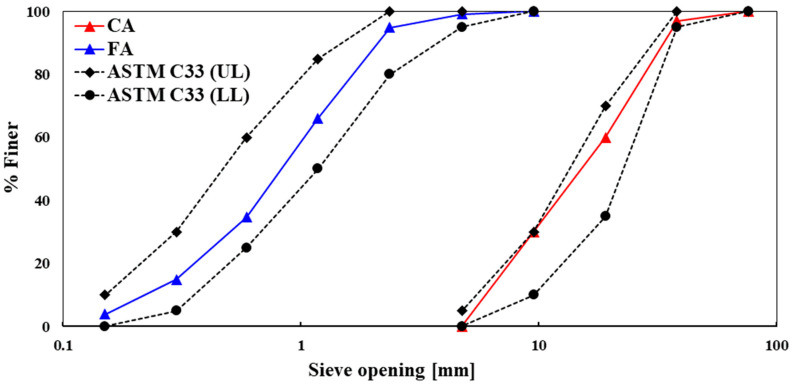
Grading curve of coarse aggregate (CA: BA and SSA) and fine aggregate (FA), and comparison with the upper limit (UL) and lower limit (LL) recommended in ASTM C33 standard [20].

The steel slag boulders were manually crushed to smaller sizes to be used as coarse aggregate in concrete (see Figure 1c). Similarly, the first-class burnt clay bricks were collected from the local market and then crushed into coarse aggregate (CA) (see Figure 1e). Thereafter, the slag and brick aggregates were sieved in the laboratory using the ASTM C136 standard sieves [19] and compared with the upper and lower limits recommended in the ASTM C33 standard [20]. To avoid the effect of grading on the mechanical and porosity performances of the concrete, similar gradation was used for both BA and SSA. The grading curves of coarse and fine aggregates are shown in Figure 2. For the physical properties of both BA and SSA, aggregates were tested for unit weight, voids, specific gravity, absorption capacity, and Los Angeles (LA) abrasion resistance as per ASTM standards. The aggregate impact value, aggregate crushing value, angularity number, flakiness index, and elongation index were measured according to BS EN 1097-3:1998 [21]. 

The chemical composition of both BA and SSA was performed by using XRF analysis. The results of the XRF analyses are presented in Table 1. The SSA contains about 26% SiO_2_, 44% Fe_2_O_3_, 4.9% Al_2_O_3_, and 4.9% CaO. By contrast, BA contains 60% SiO_2_, 14% Fe_2_O_3_, 9.9% Al_2_O_3_, and 4.1% CaO. Indeed, the oxide percentage of steel slag or brick aggregates depends on the type, source, origin, and furnace systems. It can be seen that the Fe_2_O_3_ of SSA was quite higher than the BA, which could be responsible for the higher specific gravity and density of SSA (see Table 1).

#### 2.1.2. Binder

CEM II cement was used as a binder for all mixes. The physical properties of cement such as normal consistency, initial and final setting time, as well as compressive strength were carried out as per ASTM C187 [22] ASTM C191 [23], and ASTM C109 [24], respectively. The physical and main constituents of cement are presented in Table 2. 

### 2.2. Experimental Programs, Apparatus, and Test Procedures

All the concrete specimens were produced at constant water to cement ratio (w/c) of 0.45 and cement content of 350 kg/m^3^. The experimental programs were divided into two parts: mechanical properties and porosity tests. The SSA was used to replace BA at nine different replacement levels (0%, 10%, 20%, 30%, 40%, 50%, 60%, 80%, and 100% by volume of BA). Although no chemical treatment procedure was applied, the SSA was washed with tap water before use. The mixture proportions of the concrete mixes are summarized in Table 3. The workability of the concrete mixes was investigated by measuring the slump values of fresh concrete. 

**Figure 3:**
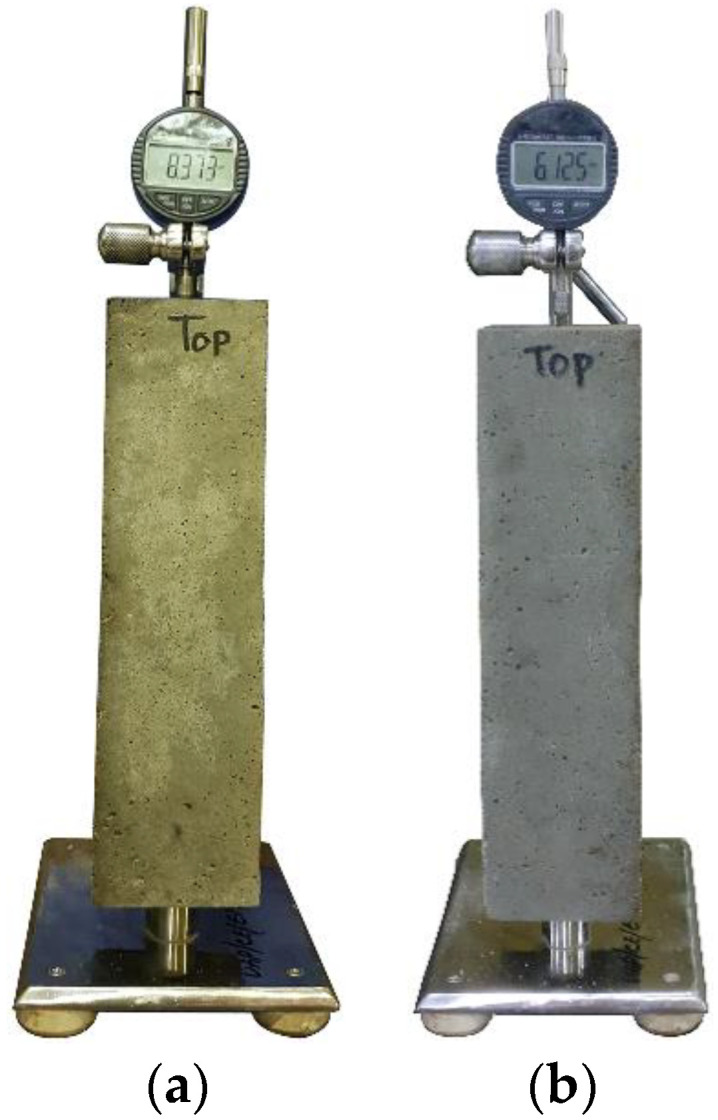
Measurement of concrete expansion made with 100% BA (**a**) and 100% SSA (**b**).

In addition, the temperature of the fresh concrete mixes was measured at all replacement levels. Since SSA is the byproduct of the steelmaking industry, the rate of expansion (length change) of the hardened concrete was monitored. Specimens (three replicates each) from three concrete mixes containing 0%, 50%, and 100% (by volume of BA) SSA were used for the investigation of expansion. The measurement of the longitudinal deformation of the specimens was conducted at different ages according to the French standard, NF P18-454 [25], using prismatic specimens (70 mm × 70 mm × 280 mm) (see Figure 3). As a reference, the first value was taken right after 24 h of casting concrete samples. A high-accuracy digital dial gauge with a precision of 0.001 mm was used to monitor the longitudinal length change.

#### 2.2.1. Mechanical Properties

A total of 216 cylindrical specimens (100 mm in diameter and 200 mm in length) for the nine different mixes was cast for compression and tensile (splitting) strength of the concrete mixes. Then, 24 h after casting, specimens were cured underwater (20 ± 2 °C) up to the day of the tests. The compressive and tensile strength tests were performed at 14, 28, 60, and 90 days to monitor the evolution of strength as per ASTM C39 [26] and ASTM 496 [27], respectively. During the compression test, the deformation of concrete specimens was measured by a strain measurement setup with dial gauges attached with an extensometer. The gauge length was 100 mm in the central part of the cylinder specimen. Three specimens were used for each curing age and each mix of both compressive and splitting tensile strength tests, and then the average strength was calculated by the arithmetic mean of three specimens. Moreover, the dry density of concrete specimens was measured on the same specimens that were used for the mechanical tests. Finally, the fractured surfaces of the specimens after compression and tension tests were studied.

#### 2.2.2. Porosity

**Figure 4:**
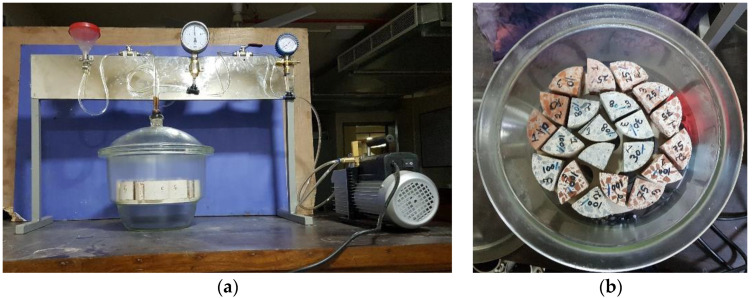
Test setup for porosity according to the French standard NF P18-459 [28] (**a**) and specimens for porosity test (**b**).

The porosity of the concrete mixes was investigated using a technique based on water absorption porosity according to the French standard, NF P18-459 [28]. The porosity test was performed using concrete specimens of a quarter-cylinder, 104 mm in diameter and 50 mm in thickness (see Figure 4). To monitor the evolution of apparent porosity as a function of time, the porosity test was conducted at 14, 28, 60, and 90 days for all the mixes. Within the scope of the study, a total of 108 concrete specimens for the nine different mixes was tested. The porosity of concrete (in %) was calculated by measuring the mass of concrete specimens in three states of saturation: (i) mass of dry ($M_{dry}$) test specimen, (ii) apparent mass of immersed ($M_{sat}^{imm})$ test specimen, and (iii) mass of soaked ($M_{sat})\;$test specimen. To do this, first, the specimens were dried in an oven at a temperature of 105 °C ± 5 °C until a constant value of mass was reached, and the mass of the dry specimen was recorded. Then, the mass of the specimen was measured in a saturated state when it was immersed in water. In this stage, a vacuum pressure of 25 mbar was attained and maintained for 4 h. After that, the specimens were immersed in water for 24 h. Finally, the saturated specimens were removed from the water and a damp cloth was used to remove excess surface water. The mass of all specimens was measured, and the apparent porosity, *Pa* (%), was computed for the three states investigated according to Equation (1).

(1) $$P_{a}=\frac{M_{sat}-M_{dry}}{M_{sat}-M_{sat}^{imm}}$$

where $M_{dry}$ is the mass of dry test specimen, $M_{sat}^{imm}$ is the apparent mass of immersed test specimen, and $M_{sat}$ indicates the mass of the soaked test specimen.

#### 2.2.3. Scanning Electron Microscopy (SEM)

Scanning electron microscopy (SEM) of the BA and SSA was performed to understand the pore structure and surface roughness of the aggregates. Indeed, pore structure and surface texture of the aggregates are very important for concrete since they influence the physical, mechanical, and durability performances of concrete. Furthermore, SEM observations were also carried out on the concrete samples to detect possible cracking at the interface between BA/SSA and cement paste. Observed cracks could be correlated to the mechanical and durability performances of concrete made with these aggregates. All SEM tests were carried out using JEOL JSM-7600F Schottky Field Emission Scanning Electron Microscope. For the investigation of the interfacial transition zone (ITZ), completely dry concrete specimens were used, which had been polished with sandpaper and then submersed in ethanol solution.

## 3. Experimental Results and Discussion

### 3.1. Physical Properties of Aggregates

The physical properties of the coarse and fine aggregates are summarized in Table 4. The specific gravity of the SSA was higher than the BA, which is believed to have been caused by its higher Fe_2_O_3_ content as shown in Table 1. Conversely, significantly lower values of LA abrasion, impact value, flakiness index, and elongation index were observed for SSA than BA, resulting in higher mechanical properties and durability of concrete made with SSA than BA (see Table 4). As regards the angularity number, SSA provides higher values than BA (9.13 for SSA and 11.20 for BA); therefore, on the one hand, SSA could reduce the workability of the concrete because of high stability due to better interlocking and friction. On the other hand, it could result in better ITZ between SSA and cement mortar, meaning higher strength and durability. Moreover, significantly lower absorption capacity was observed for SSA than BA (1% for SSA and 20% for BA). The lower absorption capacity of SSA could be explained by the impervious nature of SSA compared to BA, resulting in less permeable and durable concrete when SSA is used. 

### 3.2. Fresh Concrete Properties

The workability of different concrete mixes was evaluated by measuring the slump value of fresh concrete at the time of placing (Table 5). No segregation and bleeding were observed during and after the fresh concrete placement. It was observed that the concrete made with SSA had lower workability as compared to concrete made with BA. The slump values of concrete made with 0%, 50%, and 100% of SSA were 21.70 cm, 18.70 cm, and 16.20 cm, respectively. It was about 13% and 25% lower slump for concrete containing 50% and 100% SSA, respectively, as compared to concrete made with 100% BA. The visual observation of SSA shows that SSA was highly angular in shape with sharp edges and had a rougher surface texture than BA (see Figure 1d,f), resulting in the reduction of the flowability of concrete due to better interlocking in the mix. Similar results were found in the literature [7]. The reduction in the flowability could also be linked to the temperature of concrete at the time of placing. As shown in Table 5, as the temperature of the fresh concrete increases, the workability of the concrete decreases due to faster heat of hydration of cement paste. It was observed that the temperature of the fresh concrete at the time of placement was higher for SSA than BA. 

### 3.3. Concrete Expansion

**Figure 5:**
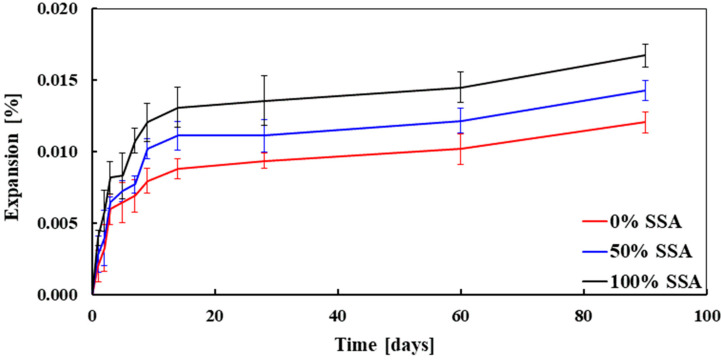
Longitudinal expansion of concrete mixes as a function of time.

The longitudinal expansion of concrete mixes as a function of time is presented in Figure 5. It was observed that the expansion of concrete made with 100% SSA was higher than the concrete made with 50% SSA and 0% SSA (100% BA). The average expansion of concrete made with 0% SSA, 50% SSA, and 100% SSA measured at 90 days was 0.012%, 0.014%, and 0.017%, respectively. These values of expansion of SSA concrete are consistent with the results found in previous studies [11,17,29]. The higher expansion of concrete made with SSA could be explained by the higher amount of calcium oxide (4.18% for BA and 4.94% for SSA, see Table 1). Several researchers reported that free calcium and magnesium oxides were the main reasons for the expansion of SSA concrete [11,17]. However, the expansion of SSA concrete was not significantly high as compared to BA concrete, which is consistent with the XRF results. In Table 1, it is reported that the calcium oxide content of SSA was very close to BA, while the magnesium oxide was much lower for SSA than BA (1.69 for BA and 0.47 for SSA, see Table 1). It is well-known that the expansion and cracking of concrete lead to loss of strength and slope of the stress–strain curve (i.e., modulus of elasticity). It should be noted that no cracking was observed at the ITZ of concrete made with SSA (see Section 3.7), and both compressive and tensile strength were also higher for SSA than BA, which is consistent with the expansion results. 

### 3.4. Dry Density of Concrete Mixes

The dry density of concrete was measured on the same concrete cylinders that were used for the compressive strength tests. The average dry density and increase in dry density of hardened concrete are presented in Table 6. As expected, with an increase in the percentage replacement of BA by SSA, the density of hardened concrete also increases. 

The average density of concrete made with 0% SSA (100% BA), 50% SSA, and 100% SSA was 2132 kg/m^3^, 2514 kg/m^3^, and 2668 kg/m^3^, respectively, at 28 days and 2145 kg/m^3^, 2539 kg/m^3^, and 2724 kg/m^3^ accordingly at 90 days. The highest density was observed for the concrete made with 100% SSA, which was about 25–27% higher for all ages than the concrete made with 100% BA (without SSA). This higher density of concrete made with SSA is directly linked with the higher specific gravity (3.24 for SSA and 2.1 for BA, see Table 4) and better interlocking between SSA than BA. The higher density of concrete made with SSA was also reported by Adegoloye et al. [11]. 

### 3.5. Mechanical Properties

#### 3.5.1. Stress–Strain Behavior of Concrete Mixes

**Figure 6:**
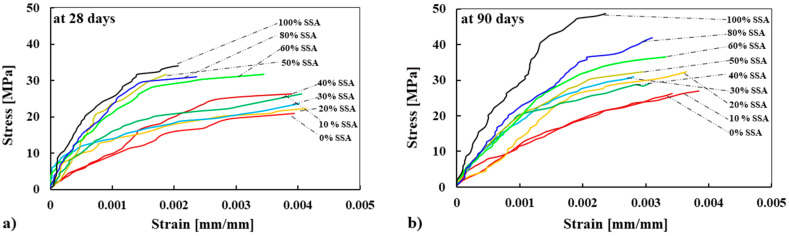
Stress–strain curves of concrete made with SSA measured at 28 days (**a**) and 90 days (**b**).

The example of the stress–strain curves in compression of all concrete mixes performed at 28 days and 90 days is plotted in Figure 6. Although three specimens for each curing age and each mix were tested in compression, the curves presented in Figure 6 are the result of one specimen for each mix. The results show that the compressive strength and the modulus of elasticity increase with the increase of replacement percentage of BA by SSA. This behavior was more pronounced when the BA was fully replaced by SSA. The strain corresponding to the compressive strength decreases with the increase of compressive strength, especially for the concrete specimens made with a higher percentage of SSA (e.g., 80% and 100% SSA). This is attributed to higher stiffness and hardness of SSA than BA, which limits the deformation (i.e., cracking on the aggregate and at ITZ) of concrete. The results also indicate that the modulus of elasticity increases over time, which could be linked to the development of strength, porosity, and ITZ around the aggregates. A similar conclusion was drawn by Qasrawi [16].

#### 3.5.2. Compressive Strength

**Figure 7:**
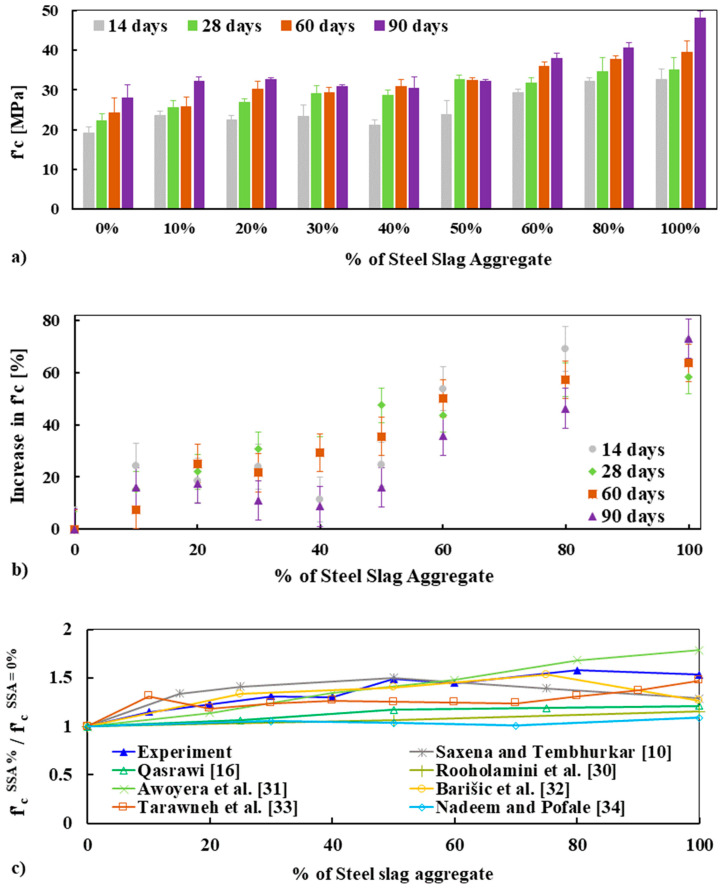
Compressive strength $f_{c}^{\prime }$ (**a**), an increase in $f_{c}^{\prime }$ as a function of SSA (**b**), and normalized $f_{c}^{\prime }$ of concrete mixes measured at 28 days were compared with other results found in the literature (**c**), respectively.

The average compressive strength of concrete mixes measured at 14, 28, 60, and 90 days is reported in Figure 7a. It is clearly shown that the concrete made with SSA has significantly higher compressive strength at all curing ages as compared to 100% BA, and this behavior was more pronounced at later ages. It is noteworthy that none of the concrete specimens made with SSA shows lower strength in compression as compared to that of 100% BA. For example, the average compressive strength of concrete made with 0%, 50%, and 100% SSA was 22.24 MPa, 32.63 MPa, and 34.98 MPa at 28 days, respectively, and 28.03 MPa, 32.18 MPa, and 48.00 MPa at 90 days accordingly (see Figure 7a and Table 7). The increase in the compressive strength of concrete mixes made with the nine different percentage replacements of BA by SSA for all curing ages was in the range of 10% to 70% of the strength of BA (see Figure 7b). The higher compressive strength of concrete made with SSA could be attributed to higher aggregate strength (LA abrasion: 19.4% for SSA and 42.7% for BA), higher crushing and impact resistance, impervious nature (i.e., lower porosity, see Figure 1d), higher angularity (angularity number: 11.20 for SSA and 9.13 for BA), and stronger bond with cement paste of SSA than BA. 

By visual inspection it was observed that the SSA was highly angular in shape, had higher rough surface texture and lower flaky particles than the BA, which is the key feature to a stronger bond between the aggregate and cement paste in the concrete matrix. These results are consistent with the results available in existing studies [11]. For a uniaxially loaded concrete, on the one hand, the micro-cracks and cracks are open parallel to the applied loading direction; on the other hand, it decreases the crack opening that is perpendicular to the load [30,31]. Generally, the cracks are more pronounced for weak ITZ and weak aggregate. Therefore, the cracks were higher in BA concrete than SSA due to its softer and porousness behavior, thus providing a weak ITZ and then lower strength. Moreover, significant lower porosity was observed for the concrete made with SSA than BA (see Section 3.6), which could also explain the higher strength of SSA concrete. 

**Figure 8:**
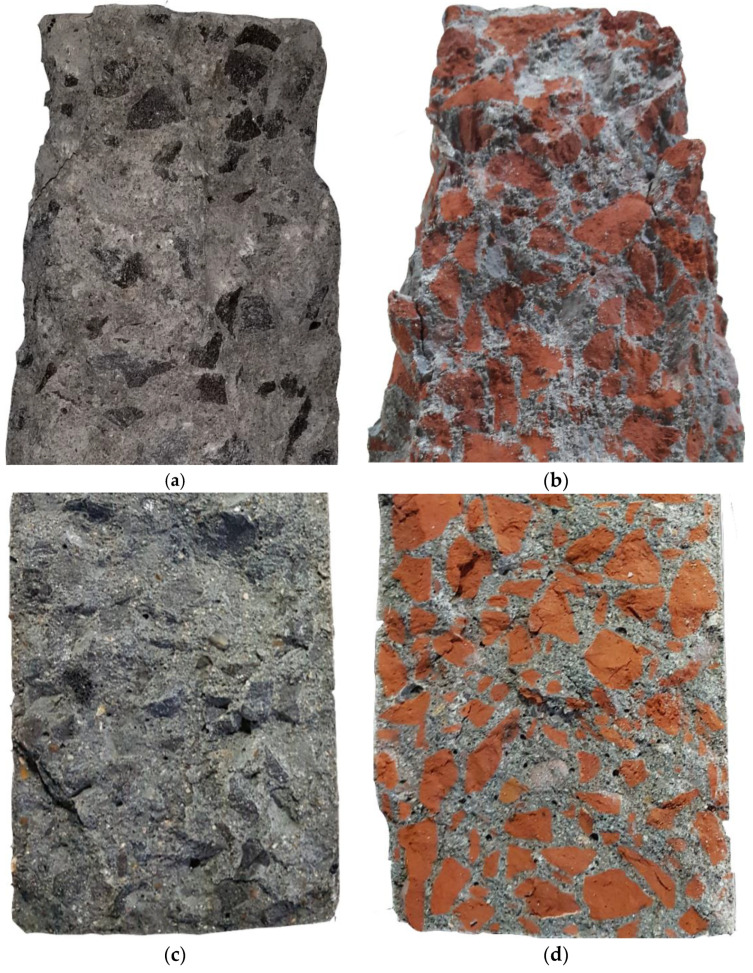
Fracture surface after compressive strength test of SSA concrete (**a**) BA concrete (**b**) and splitting tensile strength test of SSA concrete (**c**) and BA concrete (**d**) samples.

The analysis of the fractured surfaces of concrete specimens after compressive strength tests shows that the failure plane passes through the SSA (see Figure 8a) and cement mortar (i.e., combined failure). This failure pattern confirmed the stronger bond between the aggregate and cement paste. Though a similar failure pattern was observed for the concrete made with BA (see Figure 8b), the relatively low strength, higher porosity, and flakier particles of BA than SSA restricted the concrete to gain higher strength. 

To gain a deeper understanding of the role of different percentage replacement of BA by SSA on the compressive strength of concrete, normalized compressive strength at 28 days was calculated by dividing the 100% BA (SSA = 0%) concrete (i.e., $f\prime_{c}^{SSA\;\%}$/$f\prime_{c}^{SSA=0\%}$) and comparing it with the results found in the literature [10,16,32,33,34,35,36] (see Figure 7c). It was observed that the experimental results are in good agreement with the results found in the literature. Most of the researchers found similarities with this experimental work. Hence, it implies that without compromising the compressive strength, 100% BA can be replaced by SSA in concrete. 

#### 3.5.3. Tensile strength of Concrete Mixes

**Figure 9:**
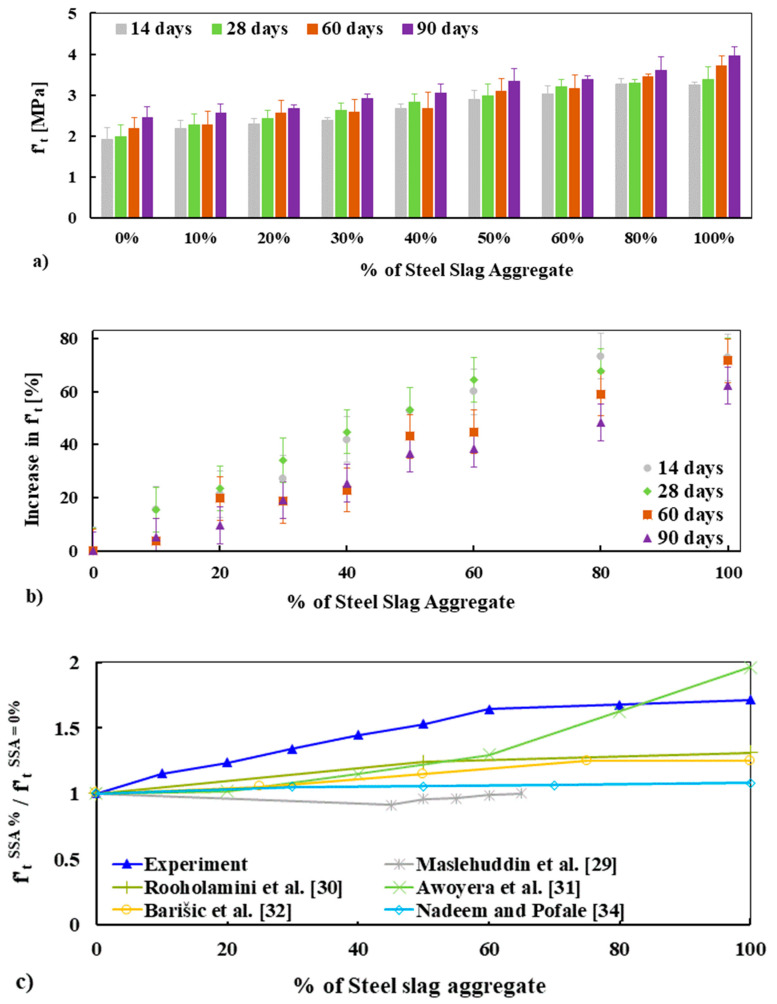
Tensile (splitting) strength: $f_{t}^{\prime }$ (**a**) and an increase in $f_{t}^{\prime }$ as a function of SSA (**b**), and normalized $f_{t}^{\prime }$ of concrete mixes measured at 28 days compared with other results found in the literature (**c**).

The splitting tensile strength of concrete mixes made with different percentage replacements of BA by SSA is presented in Figure 9a. 

It is seen that the tensile strength increases with the increasing percentage of SSA for all curing ages, which is in good agreement with the results of the compressive strength as discussed in Section 3.5.2. The splitting tensile strength of the concrete made with 100% BA (0% SSA) and 100% SSA was 2.00 MPa and 3.40 MPa, respectively, at 28 days and 2.50 MPa and 4.00 MPa accordingly at 90 days (see Table 8). The increased strength in tension of all concrete mixes made with the nine different percentage replacements of BA by SSA for all curing ages was in the range of 15% to 73% of the tensile strength of BA (see Figure 9b), which is almost similar to the strength increase in compression (see Figure 7b). These results are consistent with the results available in the existing studies [29,32,33,34,36]. Figure 9c presents the normalized splitting tensile strength at 28 days of all concrete mixes and compares with the results found in the literature. It shows that splitting tensile strength was increased with the increased amount of SSA and the results are consistent with the literature. 

As described briefly in Section 3.5.2, this higher tensile strength of concrete made with SSA could be linked to the higher strength, higher angularity, and excellent surface roughness of SSA than the BA, which ensured strong ITZ around the SSA than the BA. It is noted that the combined (cement mortar and aggregate) failure occurs for both SSA and BA concrete, which shows good adherence and cohesion between aggregates and cement mortar (see Figure 8c,d). As mentioned earlier, the lower tensile strength of BA concrete could be attributed to the relatively softer and more porous behavior of BA than SSA. In addition, SEM observations show that BA has more cracks on the aggregate as well as at the ITZ, while no cracking of this type was observed for SSA, which could be another reason for lower tensile and compressive strength of concrete made with BA than SSA (see Section 3.7).

### 3.6. Porosity of Concrete Mixes

**Figure 10:**
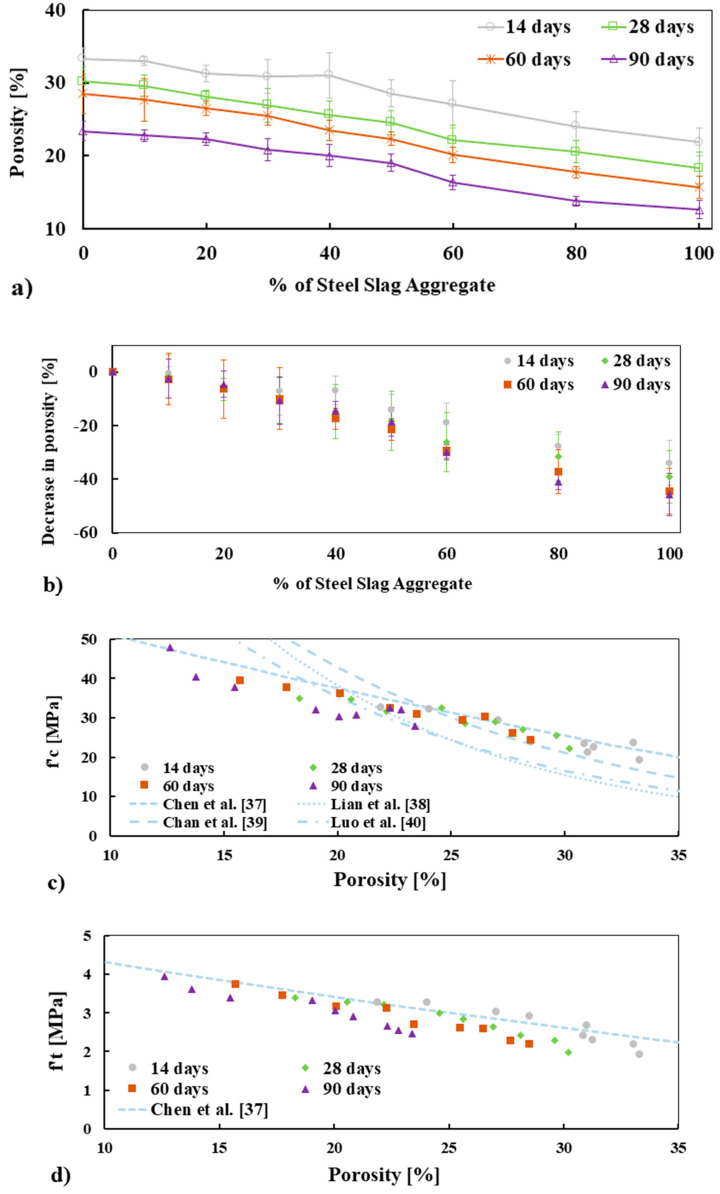
Apparent porosity (**a**), decrease in apparent porosity as a function of SSA (**b**), and the relation between $f_{c}^{\prime }$ and $f_{t}^{\prime }$ compared with different models/exponential relationships available in the literature (**c**,**d**), respectively.

To gain an understanding of the pore volume of the concrete mixes and the role played by SSA, the total porosity of concrete was measured by means of water absorption porosimetry, as reported in Figure 10a. It is seen that the porosity of the concrete mixes decreases for all ages with increasing percentage replacement of BA by SSA, see Figure 10b. This lower porosity of concrete made with SSA than BA is consistent with the higher compressive and tensile strength of concrete. The apparent porosity of the concrete made with 100% BA and 100% SSA was 30.20% and 18.30%, respectively, at 28 days and 23.40% and 12.60%, accordingly, at 90 days. The maximum decrease in porosity was observed for 100% SSA concrete, which was about 34%, 39%, 44.5%, and 45.8% at 14, 28, 60, and 90 days, respectively, lower than 100% BA concrete (see Figure 10b). This lower porosity of SSA concrete could be attributed to the dense microstructure (i.e., stronger due to lower LA abrasion, see Table 4) and lower voids (lower permeable and impermeable pores and lower connectivity of pores, see Figure 1d) of SSA than BA. 

It is well-known that porosity and permeability have a direct link [31]. Though permeability of concrete was not measured in this study, the lower porosity and better ITZ of SSA concrete imply that the permeability of SSA concrete could be lower, which could be an indication of higher durability. The relationship between porosity and both compressive and tensile strength of the concrete mixes are plotted in Figure 10c,d. The experimentally measured compressive and tensile strength of concrete mixes were compared with models/exponential relationships available in the literature [37,38,39,40]. As expected, as the porosity increases, the compressive strength decreases. It is noted that the relation between both compressive and tensile strength with the porosity of the concrete mixes for all ages are in agreement with the relationships presented in the literature (see Figure 10c,d). This relation implies that the measurement of both compressive strength and porosity are quite satisfactory.

### 3.7. Scanning Electron Microscopy (SEM) Analysis

**Figure 11:**
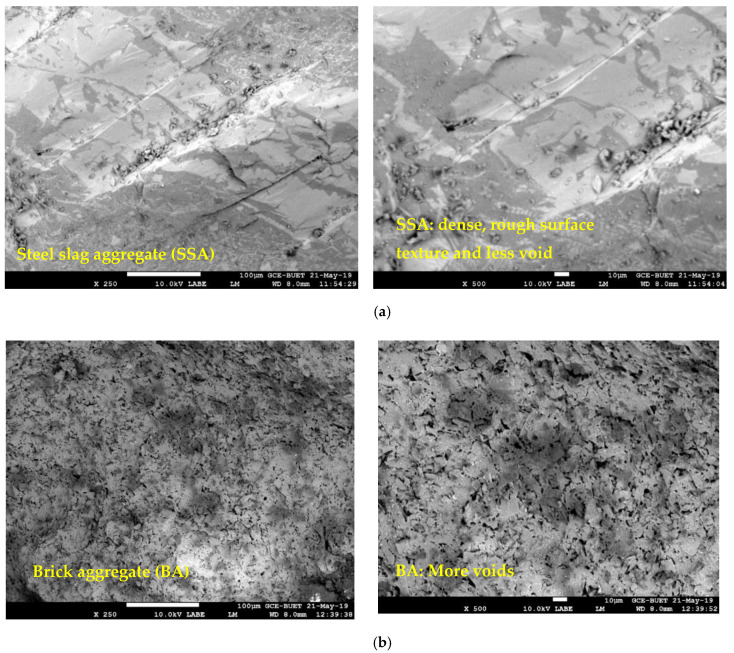

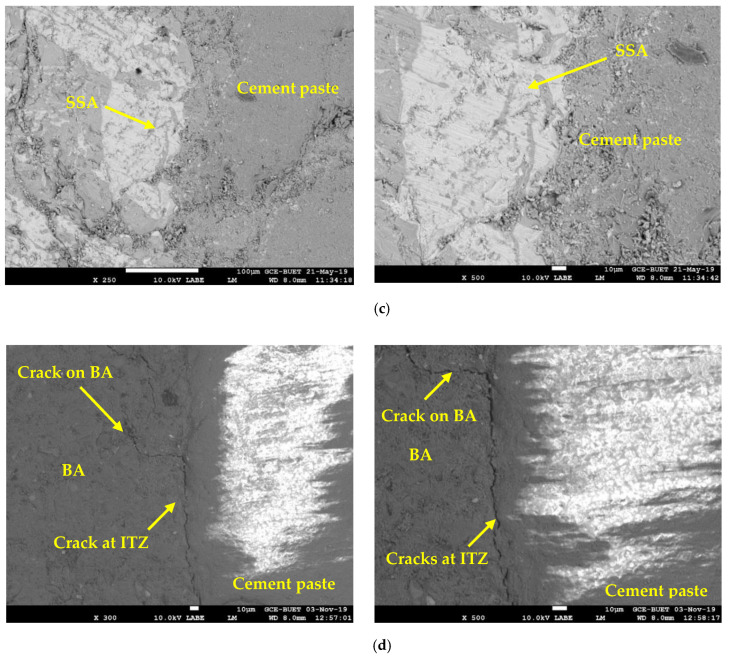
SEM images of SSA (**a**), BA (**b**), interfacial transition zone (ITZ) of concrete made with SSA (**c**) and ITZ of concrete made with BA (**d**), respectively.

The microstructure of concrete and aggregate is very important since it influences the mechanical and durability performances of concrete. The images captured by SEM analysis of SSA and BA are shown in Figure 11a,b. It is shown that the SSA was denser (i.e., strong), had a highly rough surface texture, and less voids than BA. This higher surface roughness is an important factor that affects the bonding between aggregate and cement paste (i.e., ITZ). SEM images of BA showed that it has more voids and internal cracks (see Figure 11b), while no cracking of this type was observed in SSA. These higher voids and cracks of BA affect the global strength and durability of concrete, which is in good agreement with the lower strength and higher porosity of concrete made with BA than SSA. 

To relate strength performance and possible cracks in the concrete, SEM observations were carried out on concrete samples made with 100% SSA and 100% BA at the interface between the aggregates (SSA and BA) and cement paste. The SEM images at the ITZ for both concrete mixes are presented in Figure 11c,d. A clear dense ITZ was observed around the SSA (see Figure 11c), which could be attributed to the rough surface texture of SSA (improving the bond between cement paste and SSA) that ensures better mechanical and higher durability of concrete. In contrast, it should be noted that more clear cracking through the BA and at the ITZ (see Figure 11d) and more voids (see Figure 11b) are visible in BA concrete, which has a significant effect on the global strength and durability of the concrete. The cracking in the ITZ of cement paste and BA can be explained in two ways. Firstly, the compressive strength of BA is lower than the cement paste. Typically, BA has a compressive strength of about 20 MPa, and under the compression loading test, BA reaches its ultimate load capacity level before the cement paste. Thus, the cracks can be formed in the ITZ. Lastly, the higher water absorption capacity of BA can create a weak ITZ by absorbing water from its surrounding cement paste, which may increase the unhydrated binders in BA concrete. Therefore, under the compression load, these unhydrated binders can also lead to the cracking in the ITZ. Nevertheless, these cracks and voids should be the main reason for lower compressive and tensile strength as well as higher porosity of concrete made with BA than SSA.

## 4. Concluding Remarks

In this paper, the effect of steel slag aggregate (SSA) as a substitute for conventionally used brick aggregate (BA) on the physical, mechanical, and durability (i.e., porosity) performances of concretes was investigated. It is worth noting, however, that many studies have been conducted on the mechanical properties of concrete, while comparatively, few published data are available on the durability (e.g., porosity) as well as length change. To this end, nine concrete mixes made with different percentage replacements of BA by SSA were studied. The main findings of the influence of SSA on the physical, mechanical, and porosity of concrete can be summarized as follows:

- The use of SSA as a replacement for BA in concrete shows significantly higher compressive and tensile strength, which was 73% higher when BA was fully replaced by SSA.
- Lower workability was noticed for the concrete made with SSA than BA, which could be attributed to the higher rough surface texture and higher angularity of SSA than BA as well as better interlocking, which reduces the mobility of fresh concrete.
- The concrete made with SSA exhibited higher expansion than the concrete made BA.
- A significantly lower porosity was observed for the concrete made with SSA than BA. The maximum decrease in porosity was observed when BA was fully replaced by SSA, and the decrease was 45.80% lower than BA concrete.
- A satisfactory relationship between strength (compressive and tensile) and porosity was observed, which is consistent with the literature.
- SEM images showed that SSA was denser and has a stronger ITZ, which leads to the higher strength of concrete. By contrast, BA has more voids and cracks on aggregate as well as at the ITZ, which explains the lower strength of this concrete.
- From the experimental results of the nine mixes, this study reveals that SSA can be used as a full replacement for BA since SSA is denser, less porous, higher angularity, and has excellent surface roughness, which provides better mechanical and durability performances. Furthermore, SSA concrete provides environmental solutions by reducing the dumping problem, economical, conservation of natural aggregate, and sustainable green construction material since burning brick produces a lot of CO_2_.

Future research should focus on utilizing other industrial by products and sustainable technologies such as 3D concrete printing [41,42,43] without compromising the mechanical properties required for civil applications. 

## Figures and Tables

**Table 1:** Chemical composition of brick and induction furnace steel slag.

Chemical Composition	BA (%)	SSA (%)
SiO_2_	60.43	26.18
Fe_2_O_3_	14.27	44.39
Al_2_O_3_	9.96	4.94
K_2_O	5.23	0.56
CaO	4.18	4.94
TiO_2_	1.81	1.73
MgO	1.69	0.47
Na_2_O	0.90	0.45
SO_3_	0.57	0.43
MnO	0.30	12.9
P_2_O_5_	0.24	0.08
ZnO	0.10	2.33
ZrO_2_	0.05	0.11
SrO	0.05	0.09

**Table 2:** Physical and main constituents of cement.

Properties	Observed Values
Normal consistency (%)	27.50
Initial setting time (min)	110
Final setting time (min)	360
Compressive strength (MPa) at 3, 7, 14, and 28 days	15.20, 19.50, 25.80 and 31.74
Chemical composition:	
Clinker (%)	80–94
Slag and fly ash, limestone (%)	6–20
Gypsum (%)	0–5

**Table 3:** Mixture proportion of concrete mixes (kg/m^3^).

Mix ID.	% SSA	% BA	Cement	Coarse Aggregate		Fine Aggregate	Water
				SSA	BA		
0% SSA	0	100	350	0	775	872	158
10% SSA	10	90	350	82	735	872	158
20% SSA	20	80	350	172	687	872	158
30% SSA	30	70	350	270	631	872	158
40% SSA	40	60	350	377	566	872	158
50% SSA	50	50	350	493	493	872	158
60% SSA	60	40	350	616	411	872	158
80% SSA	80	20	350	889	222	872	158
100% SSA	100	0	350	1196	0	872	158

**Table 4:** Physical properties of coarse (SSA and BA) and fine (FA) aggregates.

Properties	BA	SSA	FA
Fineness modulus	6.35	6.35	2.92
*SSD unit weight (kg/m^3^)	1120	1835	1530
Specific gravity in SSD condition	2.10	3.24	2.56
Absorption capacity (%)	20.00	1.00	3.10
Los Angeles (LA) abrasion (%)	42.70	19.40	-
Impact value (%)	28.10	8.95	-
Crushing value (%)	41.21	20.64	-
Angularity number	9.13	11.20	-
Flakiness index (%)	13.60	7.19	-
Elongation index (%)	40.67	24.19	-

Note: *SSD means saturated surface dry

**Table 5:** Slump and temperature of fresh concrete made with nine concrete mixes.

SSA	0%	10%	20%	30%	40%	50%	60%	80%	100%
Slump (cm)	21.70	20.40	21.00	20.90	19.50	18.70	18.30	16.90	16.20
Temperature (°C)	27.70	28.10	28.60	29.00	30.20	29.80	30.40	30.60	30.90

**Table 6:** The average dry density (ρ) and the increase in density of hardened concrete mixes.

Age	SSA	0%	10%	20%	30%	40%	50%	60%	80%	100%
14 days	ρ (Kg/m^3^)	2078	2290	2405	2378	2429	2494	2540	2591	2633
	Increase (%)	0.00	10.20	15.80	14.50	16.90	20.0	22.30	24.70	26.70
28 days	ρ (Kg/m^3^)	2132	2299	2351	2391	2439	2514	2555	2634	2668
	Increase (%)	0.00	7.90	10.30	12.20	14.40	18.00	19.90	23.60	25.20
60 days	ρ (Kg/m^3^)	2117	2320	2373	2401	2465	2531	2571	2628	2691
	Increase (%)	0.00	9.60	12.10	13.40	16.40	19.60	21.50	24.20	27.10
90 days	ρ (Kg/m^3^)	2145	2321	2385	2455	2487	2539	2595	2658	2724
	Increase (%)	0.00	8.20	11.20	14.50	15.90	18.40	21.00	23.90	27.00

**Table 7:** The average compressive strength ($f_{c}^{\prime }$) and coefficient of variation (CoV) of concrete.

Age	SSA	0%	10%	20%	30%	40%	50%	60%	80%	100%
14 days	$f_{c}^{\prime }$ (MPa)	19.08	23.60	22.47	23.37	21.11	23.82	29.24	32.09	32.63
	CoV (%)	8.94	4.98	4.61	11.72	6.68	14.60	3.35	2.88	8.39
28 days	$f_{c}^{\prime }$ (MPa)	22.24	25.63	26.98	29.02	28.57	32.63	31.73	34.67	34.98
	CoV (%)	8.06	7.00	2.51	7.01	4.94	3.17	4.27	9.84	9.03
60 days	$f_{c}^{\prime }$ (MPa)	24.30	25.85	30.15	29.24	30.83	32.41	36.02	37.60	39.41
	CoV (%)	15.13	9.21	6.87	4.82	5.53	2.09	2.87	2.75	7.16
90 days	$f_{c}^{\prime }$ (MPa)	28.03	32.18	32.63	30.83	30.37	32.18	37.83	40.54	47.99
	CoV (%)	11.63	3.22	1.20	1.27	9.73	1.22	3.58	3.34	3.74

**Table 8:** The average tensile strength ($f_{t}^{\prime }$ ) and coefficient of variation (CoV) of concrete.

Age	SSA	0%	10%	20%	30%	40%	50%	60%	80%	100%
14 days	$f_{t}^{\prime }$ (MPa)	1.91	2.19	2.29	2.39	2.67	2.90	3.02	3.27	3.26
	CoV (%)	15.28	9.48	6.36	2.31	4.29	7.21	6.40	4.39	1.49
28 days	$f_{t}^{\prime }$ (MPa)	1.98	2.28	2.42	2.63	2.83	2.99	3.21	3.29	3.38
	CoV (%)	14.56	11.70	8.63	6.88	7.02	9.09	5.51	2.79	9.04
60 days	$f_{t}^{\prime }$ (MPa)	2.19	2.27	2.57	2.59	2.68	3.10	3.15	3.44	3.72
	CoV (%)	11.68	14.99	12.04	12.13	14.83	9.55	10.64	2.45	6.45
90 days	$f_{t}^{\prime }$ (MPa)	2.46	2.56	2.67	2.91	3.06	3.33	3.38	3.61	3.96
	CoV (%)	10.04	8.83	3.57	3.85	6.83	9.43	2.37	9.18	5.71
